# A Case of Recurrent Cryptococcal Meningoencephalitis in an Immunocompetent Female

**DOI:** 10.1155/2014/407348

**Published:** 2014-07-15

**Authors:** Negin Niknam, Negar Niknam, Kola Dushaj, Erfidia Restrepo

**Affiliations:** Department of Medicine, Icahn School of Medicine at Mount Sinai, Queens Hospital Center, Jamaica, NY 11432, USA

## Abstract

*Cryptococcus neoformans* is commonly associated with meningoencephalitis in immunocompromised patients and occasionally in apparently healthy individuals. Duration and regimen of antifungal treatment vary depending on the nature of the host and extent of disease and CNS shunts are placed in persistently elevated intracranial pressures. Recurrence of infection after initial treatment is not uncommon in HIV positive patients, Kaya et al. (2012) and Illnait-zaragozí et al. (2010). We describe a 39-year-old immunocompetent female that presented with neurologic deficits and increased intracranial pressure (ICP) due to cryptococcal meningoencephalitis that had a complicated course with drug induced hepatitis and persistently increased ICP that ultimately required shunt placement and presented again with relapse of cryptococcal meningoencephalitis after completion of antifungal treatment. Our case shows that recurrent cryptococcal meningitis can be seen in immunocompetent patients due to prolonged placement of CNS shunt and suggests that shunts should be removed after resolution of meningitis.

## 1. Introduction

Cryptococcal meningoencephalitis is an important opportunistic infection in AIDS or immunosuppressed patients. The most common forms of immunosuppression other than HIV include glucocorticoid therapy, solid organ transplantation, cancer (particularly hematologic malignancy), idiopathic CD4 lymphopenia, and other conditions such as sarcoidosis and hepatic failure [1, 2]. It can lead to significant neurological deficits as a result of increased intracranial pressure and edema. We present a 39-year-old immunocompetent female with no significant past medical history with recurrent cryptococcal meningoencephalitis.

## 2. Case Description 

39-year-old female with no significant past medical history presented with a 3-week headache accompanied with auditory and visual disturbances. The patient was Born in Guyana, has since been living in the Bahamas, and came to the United States 3 weeks prior to presentation. On initial examination, the patient was vitally stable with no fever and in no distress and no nuchal rigidity, photophobia, or rash, with an unremarkable neurologic examination. Initial labs revealed a mild leukocytosis (WBC 11, 100/mL), normocytic anemia, mild hyponatraemia (133 mEq/L), and hypokalemia (3.2 mEq/L) with normal renal function. Noncontrast computed tomography (CT) of the head did not reveal any acute hemorrhage, infarct, or midline shift. Patient was initially admitted with a working diagnosis of complicated migraine. EEG did not reveal any epileptiform activity, and repeat Head CT was unchanged from the prior.

The following day, the patient developed right-sided weakness, ptosis, nuchal rigidity, photophobia, deteriorating vision, and hearing loss. Lumbar puncture was performed and revealed an opening pressure of 570 mm H_2_O. CSF analysis revealed WBC of 91, lymphocytes of 75%, protein 76, and normal glucose and VDRL was negative, myelin basic protein was negative, cryptococcal Ag titer was 1 : 2840, and CSF culture was positive for* Cryptococcus neoformans. *Organism was isolated using a VITEK 2 colorimetric yeast identification card; however, sensitivities were not performed as they are not routinely done at our facility. As the patient showed improvement initially to treatment and follow-up cultures were negative, resistance to medication was unlikely.

Brain MRI was performed and revealed several areas of T2 flair signal abnormality with no postcontrast enhancement. HIV testing was negative. CD4 was 981, CD8 was 616, CD3 was 1597, and HTLV 1 was negative. Brain stem evoked auditory response showed bilateral 8th nerve dysfunction. Ophthalmology evaluation revealed severe papilledema, decreased visual acuity, and left 6th nerve partial palsy.

Patient was started on Amphotericin B and Flucytosine and serial lumbar punctures were performed that showed persistently elevated opening pressures. However, as the neurologic symptoms did not improve, dexamethasone was started and, ultimately, a lumboperitoneal shunt was indicated and placed.

After shunt placement, the patient improved significantly. The papillary edema, 8th and 6th nerve dysfunction, and right-sided weakness resolved. Patient received Amphotericin B and Flucytosine for 5 weeks and was discharged to complete an eight-week course of oral Fluconazole. However, Fluconazole was discontinued for drug induced hepatitis and patient was started on Amphotericin B and Flucytosine again for a total of two months. Patient had two negative CSF cultures and was completely asymptomatic after completion of antifungal course.

**Figure 1:**
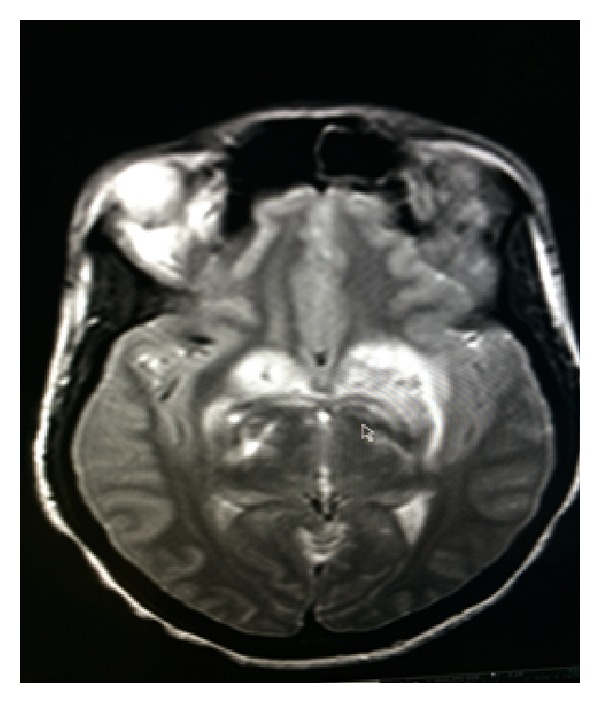
MRI-T2-Axial View: vasogenic edema of basal ganglia.

**Figure 2:**
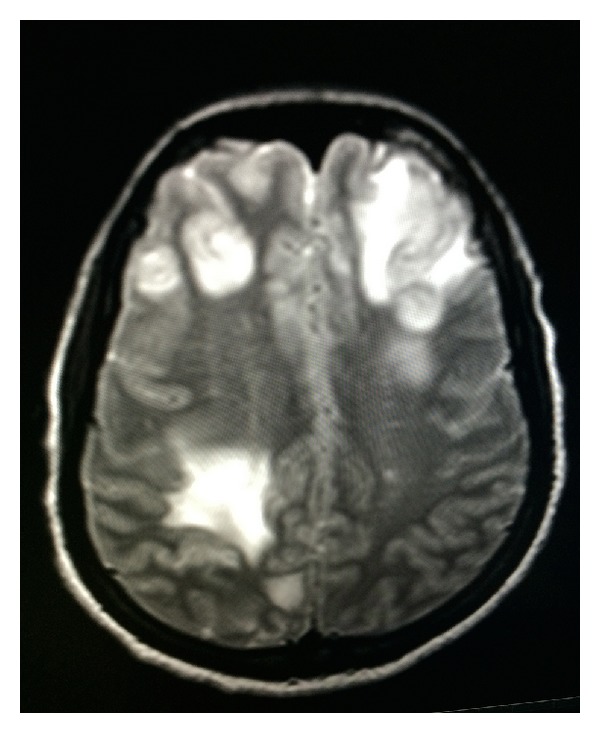
MRI-T2-Axial View: left frontal and right parietal.

**Figure 3:**
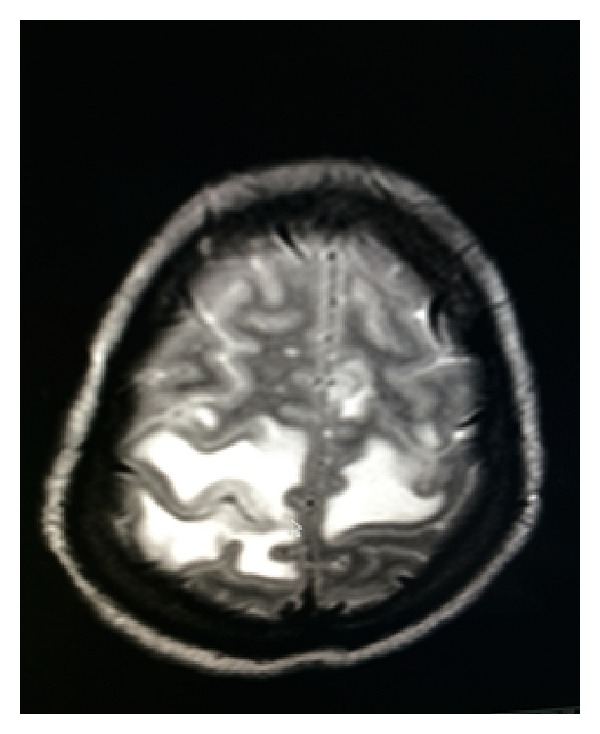
MRI-T2-Axial View: bilateral parietal vasogenic edema.

While off treatment, the patient presented five weeks later with headache, somnolence, unsteady gait, and left upper extremity weakness for one week. Brain MRI showed diffuse vasogenic edema with mass effect and irregular nodular enhancement in left frontal and parietal areas (Figures 1, 2, and 3). Patient was restarted on Liposomal Amphotericin B and Flucytosine. Dexamethasone and Mannitol were started for the vasogenic edema and Metronidazole, Vancomycin, and Ceftriaxone were started for possible shunt related infection.

Lumboperitoneal shunt was removed and CSF analysis at that time revealed WBC of 40, segmented neutrophils of 1, lymphocytes of 97, monocytes of 2, and RBC of 3 and protein was 117, glucose was 64, gram stain was negative, acid fast testing was negative, and India ink preparation was positive for encapsulated yeast. Given that the India ink preparation of the CSF fluid has remained positive, recurrence of infection was suspected. However, CSF culture and shunt tip were negative for fungal growth.

Vancomycin, Metronidazole, and Ceftriaxone were discontinued. Repeat Head CT showed improving vasogenic edema. Patient received Amphotericin B and Flucytosine for 2 months with complete resolution of neurologic symptoms. Further workup failed to show any malignancy, immunosuppression, or underlying conditions.

## 3. Discussion 

Cryptococcal meningoencephalitis is mostly seen in patients with AIDS and immunosuppression. However, in 30% of cases, no underlying condition was found [1].

*Cryptococcus neoformans* causes infection following inhalation through the respiratory tract. The organism disseminates hematogenously and has a propensity to localize to the central nervous system (CNS). Production of mannitol contributes to brain edema and inhibition of phagocyte function. Elevated intracranial pressure occurs in almost 70% of AIDS patients with cryptococcal meningoencephalitis but is observed less frequently in non-HIV-infected patients [2].

Clinical presentation of cryptococcal meningoencephalitis in HIV seronegative patients is variable. Some patients have symptoms for up to several months prior to diagnosis, whereas others present with an acute illness of only a few days. Most patients present with signs and symptoms of subacute meningoencephalitis. Regimen and duration of antifungal treatment usually depend on underlying immunosuppression [3, 4].

Therapeutic lumbar drainage should be repeated daily in the setting of clinical symptoms and persistent pressure elevation ≥25 cm H_2_O of CSF until it is stabilized for >2 days. Temporary percutaneous lumbar drains or ventriculostomy may be appropriate for patients who require repeated lumbar punctures. Ventriculoperitoneal shunting may be warranted if the patient is receiving appropriate antifungal therapy, and more conservative measures to control increased intracranial pressure have failed [5, 6]. Although CNS shunts have been very effective in decreasing the intracranial pressure and hydrocephalus in cryptococcal meningitis, they can lead to shunt infection in immunosuppression patients or relapse of cryptococcal meningoencephalitis, which suggests that CNS shunts should be removed after resolution of meningitis.

*Cryptococcus neoformans* has been known to suppress cell-mediated immunity and lead to immune reconstitution syndrome (IRS). In addition to antigen-specific responses, C neoformans is capable of eliciting an innate T-lymphocyte response as a mitogen. Mitogen-activated T cells could potentially lead to potent proinflammatory responses and, therefore, IRS [7]. The patient presented in this case report does not meet criteria for IRS as her symptoms recurred while off treatment for 5 weeks. Furthermore, the India ink preparation was positive.
